# Examining Boards and Their Selection

**Published:** 1896-11

**Authors:** 


### Editorial.

### EXAMINING BOARDS AND THEIR SELECTION.

The difficulties surrounding pioneer work in any direction, whether this be in levelling the virgin forest or in opening up new avenues of thought, have been dwelt upon in themes from time immemorial. It is certainly a law of development, from the atom to the formation of a world, that time is required to reach an approximation to perfection. This being true, criticism may be out of place where crudities are discovered in any given line of work, and it would, perhaps, be better to advise a quiet waiting for the regular processes of growth before exhibiting impatience.

While this thought should have great force in considering any subject, it should not be permitted to dominate freedom of utterance, for this, in itself, becomes a pronounced factor in progress.

With this view and with a desire to aid in arriving at a more perfected standard in the selection of members of State boards, discussion is not only profitable, but the only method to improve upon the crudities very apparent in the laws at present in force in the several States for the government of the practice of dentistry.

The departure from the old plan of unlimited freedom of practice has been along a somewhat thorny path. It could not have been expected that the American spirit, naturally antagonistic to the enslavement of law, would take kindly to legislative interference. This opposition has not been confined to a class always resistant to law, but is found largely among those who have had most to do with the training of young men for the profession. The reason for this opposition is readily explained. It has not arisen from opposition to legislative enactments so much as to the fact that educators felt that the laws, as prepared in the earlier periods, were framed by men with, no doubt, excellent motives, but lacking that experience in the needs of student life that can only be acquired by long training. The result was that almost every State law has been amended in repeated sessions of legislatures to an extent that very few, even in the State in which they are domiciled, can tell exactly what the law requires. This is an exceedingly unpleasant state of affairs and has led to misunderstandings and open conflict with State boards.

It is time some action was being taken looking not so much to a modification of the laws of the several States as to the more perfect systematizing of these, and a unification of the statutes so that uniformity of action will prevail. It is in the last degree an absurdity that a nation claiming a central power controlling all its members, that any one or all of these should be permitted to enact laws antagonizing every other member of the Confederacy, and yet this is exactly what has been done in the past and is being done in the present. A graduate to-day, in most cases, cannot cross over the border of an adjoining State and practise on his diploma, although that diploma may have received the highest indorsment of his school and the Board of Examiners of his State.

The origin of this State antagonism has had its inception in pure selfishness, a desire to bar out and make it almost impossible to practise except within prescribed boundaries.

That these enactments are contrary to the Constitution of the United States must be clear to every intelligent reader of that document. That this is the legal view is evident from the formation of the Interstate Commission to prevent discrimination in traffic between States.

A diploma from a recognized school of dental instruction, fortified by a re-examination of the State boards, should be admissible in any State of this Union, and the time has arrived when some effort must be made to effect this desirable object.

The question has been discussed for a sufficient period, and it is to be regretted that the wise suggestions of President Crawford at the last meeting of the American Dental Association met with no adequate response from those in authority.

These suggestions should form a basis of action and the initiative might very properly be taken by the National Association of Examiners. The following are the salient points in Dr. Crawford's address:

"*First.* I will say that any enactment placed upon the statute books of our common country, or any of the independent States of our common country, should rest upon the immutable principle of equal justice to all and exclusive privileges to none.

"*Secondly.* All laws that are intended to control the conduct of an individual or individuals in regard to any definite and universally uniform question in all the States should be absolutely the same in all particulars.

"*Thirdly.* All such laws should be plain and simple in their construction that the humblest citizen could comprehend them, and, if necessary, administer the same. . . .

"*Fourthly.* The penal feature should be so well marked and imperative that none would dare disregard, only at their peril. . . .

"*Fifthly.* Each State should have the same enactments controlling the matter of dental education, making the requirements for graduation uniform in every particular, thus holding dental colleges up to a higher and better standard.

"*Sixthly.* The first registration of an individual to practise should be made within twelve months after receiving a diploma from a regular dental institution of learning. The second or subsequent registrations in any other State of the Union should depend upon the presentation of a certificate from the State Board of Dental Examiners that the individual held a diploma from a reputable dental college."

To carry out these views the president suggested the appointment of a committee to consist of one member from each State appointed by the American Dental Association, National Association of Dental Examiners, and the National Association of Dental Faculties, "to be known as a general legislative committee, whose duty it shall be to formulate and draft such a uniform law as could not fail to be satisfactory to all the States."

These suggestions are specially called to the attention of the dental profession as a method of extrication from the present discreditable tangle of legislative enactments which have become a serious burden.

No reason exists why the National Association of Dental Examiners should not ask for the appointment of a national committee, as recommended, and it would seem that this might very properly be done by any national organization. A year must elapse before these can meet, and in the mean time the matter should be thoroughly canvassed in that that this exceedingly incongruous state of affairs may be changed.

In this connection the methods adopted in the appointment of members of State examining boards should be considered. In a former article these methods were criticised as unworthy an educated body, but no change has been made, nor are we aware that any effort at reform, in this direction, has been attempted. Politics governs with the same powerful influence as heretofore. The governor in some of the States still appoints whom he pleases, irrespective of ability, and in others the State society presumably sends up its best men to receive the endorsement of that functionary. This latter method would seem to be the proper channel through which these appointments should come, but, unfortunately, from recent experiences, this has its objections, and it is very evident that some other method of forming State boards must be devised if these are to continue to hold the respect of the dental profession, for, as at present constituted, the entire system is in danger of sinking into deserved contempt.

The colleges of the country have borne much in this direction, in the hope that the time would come when the evils of the present crude system would be eliminated, but instead of improvement there seems to be a decided change for the worse, and these institutions find themselves in a maze of difficulty, from which extrication seems impossible except through radical measures.

The recent proceedings of the Pennsylvania State Society, so severely criticised in our last number by the Odontological Society of Pennsylvania, is but another evidence that even State associations are not to be depended upon in this matter. The action taken there deserves and should receive the severest condemnation, and it must remain a surprise to the profession in Pennsylvania that the council of the State society has permitted months to elapse without, as far as we are aware, any attempt at investigation. It is not sufficient that the gentlemen elected to fill the vacancies upon the State board, and who are above reproach, should have resigned, the demand being for light upon the entire disagreeable subject. If the fell spirit of caucus and intrigue can invade a State organization, then all confidence in the present plan of forming State boards must be shattered.

It would seem from the distracted condition of professional legislation in this country that nothing short of an entire change of front in relation to it will be of any avail. The measures to meet this in our complicated system of government may not be clear or devoid of difficulties to any single mind, but it is hoped that out of the present confusion there may arise an intelligent arrangement whereby something more satisfactory than the present methods can be devised. While these are continued there will be constant inharmony and antagonisms of a serious character. To obviate this there must be a unification of the laws, and those selected to enforce these must be above reproach, and, further, all appointments must be removed from political influences.

